# Versatile wastewater monitoring of pathogens and antimicrobial resistance enabled by metatranscriptomics and long-read metagenomics

**DOI:** 10.21203/rs.3.rs-7492978/v1

**Published:** 2025-09-10

**Authors:** Rob Knight, M. Omar Din, Rodolfo Salido, Gillian Wright, Caitriona Brennan, Madison Ambre, Lauren Hansen, Tara Boyer, Jennifer Cao, Renee Oles, Lucas Patel, Yuhan Weng, Daniel McDonald, Shrikant Bhute, Grace Solini, Smruthi Karthikeyan, Greg Humphrey, Peter DeHoff, Sarah Kralicek, Joshua Levy, Mark Zeller, Gail Hecht, Louise Laurent, Gene Yeo, Kristian Andersen, Andrew Bartko

**Affiliations:** University of California San Diego:Dpt of Pediatrics;Dpt of Computer Science and Engineering;Shu Chien-Gene Lay Dpt of Bioengineering;Halicioglu Data Science Inst;Center for Microbiome Innovation; UCSD; University of California San Diego; University of California, San Diego; University of California San Diego; University of California San Diego; University of California San Diego; University of California, San Diego; University of California, San Diego; University of California, San Diego; University of California, San Diego; University of California, San Diego; University of California San Diego; University of California, San Diego; California Institute of Technology; Caltech; University of California at San Diego; University of California, San Diego; Midwestern University; The Scripps Research Institute; Department of Immunology and Microbiology, The Scripps Research Institute; Loyola University Medical Center; University of California, San Diego; University of California, San Diego; Department of Immunology and Microbiology The Scripps Research Institute La Jolla CA USA; University of California San Diego

## Abstract

Widespread interest in the development of population-wide pathogen and antimicrobial resistance (AMR) monitoring has revealed wastewater’s microbial footprint as a marker of public health. Near-source wastewater remains a difficult sample type for microbiome analyses but represents a closer link to human health than the downstream products of its treatment. Few studies integrate methods for non-targeted monitoring applications, and critically, current methods cannot connect AMR genes to species, nor resolve full genomes. We address these challenges by developing a pipeline that enables untargeted metagenomics, metatranscriptomics, and novel long-read metagenomics (LRG). We achieve untargeted pathogen detection, limited by highly abundant resident species, while retaining microbial information with near-source sampling. Furthermore, LRG identifies antibiotic resistance gene-containing microbes and enables assembly of culture-independent genomes with previously unreported AMR genes. We establish an integrated approach to broadly monitor pathogens in wastewater, while demonstrating the importance of LRG to illuminate microbial AMR at the species level.

## INTRODUCTION

Wastewater contains a reservoir of microbes derived from human activity, distributed across specific urban areas^1^. Accordingly, it contains a wealth of information that is relevant to public health, particularly regarding the presence of human pathogens, which are then used as a proxy for population-wide disease activity^2,3^. The use of wastewater to determine the presence of human pathogens has a long historical precedent dating back to the early-mid 20th century, when the existence of poliovirus in wastewater was established in the United States^4,5^. Global interest for pathogen monitoring in wastewater rapidly emerged from the SARS-CoV-2 pandemic, resulting in substantial efforts to test nucleic acid-based approaches to detect numerous diverse human pathogens^6–8^.

Microbiome approaches, such as untargeted sequencing through metagenomics and metatranscriptomics, have emerged as promising avenues to broadly capture information about which pathogens are present and the diversity of antimicrobial resistance genes within the sample^9–12^. Prior studies have expanded our understanding of what these technologies can achieve, but no integrated workflow exists for application and assessment. Additionally, few studies have explored the applicability of omics approaches to detect specific pathogens against a complex background, particularly emerging bacterial or fungal pathogens, or connecting AMR genes to particular microbial species^13–19^. This information is crucial for real-world application as related microbes may not be linked to disease but rather represent a baseline influenced by environmental and human factors. Furthermore, a limitation of short read sequence analysis is the lack of sequence context causing difficulty in linking antimicrobial resistance (AMR) genes to specific microbes. Thus, it is critical for human health to overcome these challenges given that wastewater represents a reservoir for AMR activity and the rise of the ‘silent pandemic’ of AMR^20–23^.

To address these gaps, we employed a wastewater processing strategy to prepare both metagenomic and metatranscriptomic samples in parallel^24^. While prior efforts have investigated wastewater samples across treatment stages, particularly activated sludge^25^, here we sought to investigate the differences between near-source and treatment plant influent sampling. We hypothesized that the sampling source would affect the amenability of specific pathogen monitoring. Informed by the metagenomic sequencing data, we selected three known and emerging pathogens which were present in wastewater at different abundance levels and introduced them as extracted nucleic acid spike-ins into wastewater samples to determine the ability of either omics approach to perform robust detection against various levels of background. We tested different computational strategies to overcome challenges in detection, including genome coverage determination and read mapping to specific pathogenic loci. We also tested whether DNA produced from this process was amenable to long-read metagenomics for further species and strain-level analyses. We demonstrate that sequencing produced by our long-read metagenomics protocol identifies microbial species carrying antibiotic resistance genes (ARGs) in wastewater using recently developed long-read tools. Furthermore, we can identify strain level AMR information and novel lineages using high-quality and complete long-read metagenomic assembled genomes from long-read sequencing.

## RESULTS

### Near-source wastewater and treatment plant influent exhibit distinct microbial diversity patterns

**Figure 1:**
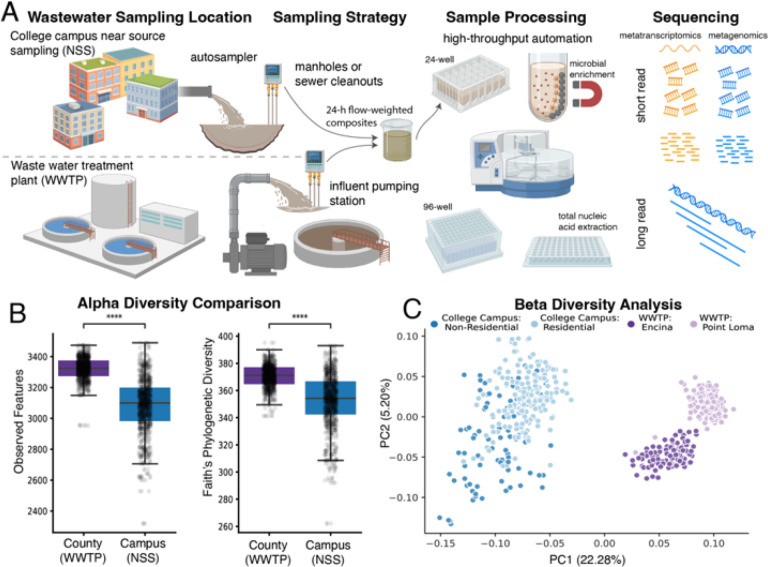
Community-level analysis reveals distinct microbial diversity patterns in near-source and wastewater treatment plant samples. **A) Automated wastewater processing workflow.** 24-hour flow-weighted wastewater composites collected from near-source sampling (NSS, *n = 285*) at campus manholes or sewer cleanouts, as well as at influent pumping stations of wastewater treatment plants (WWTPs *n = 286*; Point Loma and Encina facilities). Samples were processed in automation-compatible microplates with stepwise increases in sample density, followed by total nucleic acid extraction for parallel metagenomic and metatranscriptomic library preparation from a single sample. **B) Alpha diversity comparisons.** Boxplots display distribution of alpha diversity metrics of uniquely Observed Features and Faith’s Phylogenetic Diversity from metagenomic reads (Mann-Whitney-Wilcoxon two-sided test with Benjamini-Hochberg correction, **** denotes P ≤ 1× 10^−4^). **C) Beta diversity and community structure.** Principal coordinate analysis (PCoA) of unweighted UniFrac distances illustrate differences in metagenomic community composition between NSS samples from the UC San Diego campus compared to WWTP samples from San Diego County (PERMANOVA P = 0.001). WWTP microbial communities further segregated by county catchment area, while NSS communities differed between residential and non-residential buildings. Panel A is created in BioRender. Din, O. (2025) https://BioRender.com/.

Near-source sampling (NSS, *n = 285*) of wastewater was conducted across multiple University of California, San Diego (UC San Diego) campus buildings using autosamplers placed at manholes or sewer cleanouts. In parallel, wastewater treatment plant (WWTP, *n = 286*) samples were collected at the influent pumping stations of the Point Loma and Encina facilities, which serve San Diego County (with catchment sizes serving 2.3 million and 380 thousand people respectively). Sampling exclusively at influent points ensured the analysis of untreated wastewater, minimizing microbial diversity shifts associated with treatment processes and preserving the microbial composition representative of contributing populations (Fig. 1A). Wastewater composites were collected over 24-hour periods using flow-weighted sampling on each sampling day. All samples were collected contemporaneously (between April and May of 2023), reflecting a temporal range of signal proximal to human activity (NSS) as well as an aggregate of big populations (WWTP) representing the geospatial region of San Diego.

Collected wastewater underwent microbial enrichment via magnetic bead-based affinity capture in 24-well microplates. Enriched material was then consolidated into 96-well plates for total nucleic acid extraction, yielding input for both high throughput metagenomic and metatranscriptomic library preparation from a single sample. To support large-scale deployment, we developed a fully automated, end-to-end workflow that streamlines processing and allows cost-effective expansion of untargeted sequencing by reducing manual labor, since this is an approach that is otherwise substantially more labor and cost intensive than PCR-based detection methods, or hybrid capture panels.

To assess differences in microbial diversity between NSS (Campus) and WWTP (County) sources, we analyzed both alpha diversity (within-sample) and beta diversity (between-samples) metrics derived from operational genomic units (OGU) resulting from analysis of metagenomic short reads via Woltka^26^ and the Web of Life (release 2)^27^. Only OGUs with ≥1% genome coverage breadth were retained for community analysis. WWTP samples exhibited significantly higher median alpha diversity than NSS samples, measured by uniquely observed features (Mann-Whitney-Wilcoxon two-sided test with Benjamini-Hochberg correction, P = 3.537 × 10^−^62, U = 7.356 × 10^4^) and Faith’s Phylogenetic Diversity (P = 1.183 × 10^−23^, U = 6.052 × 10^4^) (Fig. 1B). In contrast, NSS samples displayed greater variance in alpha diversity (F-test, alpha = 0.001, Degree of Freedom between groups = 1, Degree of Freedom within groups = 569, F-value = 424.75, F-critical: 15.35), suggesting that fine-scale microbial information directly related to human activity may be lost or obscured by aggregation at the county level.

When samples were aggregated by source (Campus vs County) and classified at the species and phylum level, an intersection analysis revealed that NSS had the same breadth of unique species and phyla compared to WWTP samples covering the entire county (Extended Data Fig. 1A). This pattern was consistent when accounting for evolutionary relationships among taxa using Faith’s Phylogenetic Diversity (PD) (Extended Data Fig. 1B). Beta diversity analysis based on unweighted UniFrac distances demonstrated clear separation between NSS and WWTP communities, with further segregation observed between WWTP catchment areas and between campus residential and non-residential sources (Fig. 1C, PERMANOVA all-on-all, P = 0.001, pseudo-F = 74.29, pairwise group comparisons in Supplementary Table 1). Inter-sample dissimilarity and community composition variance was also higher in NSS wastewater, reflected by greater group dispersion in PCoA space as quantified by convex hull analysis (Extended Data Fig. 1C, Supplementary Table 2).

Taken together, these results indicate that near-source wastewater exhibits lower per-sample alpha diversity on average but captures similar aggregate diversity across samples as WWTP samples, and additionally possesses higher variability in community composition. These results suggest that near-source wastewater represents microbial communities directly related to human activity distinctly from downstream samples.

### Metagenomic monitoring of specific pathogens can be achieved by tracking genomic mapping

**Figure 2:**
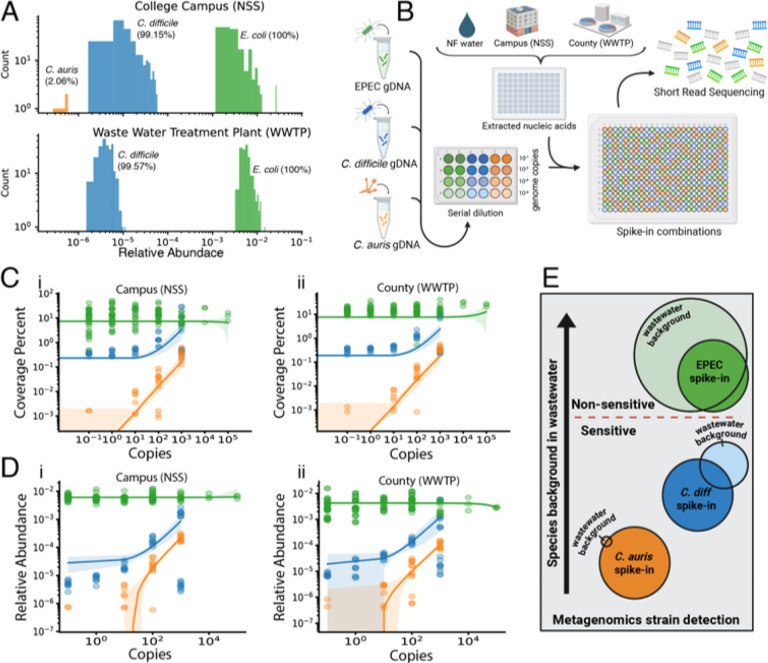
Pathogen detection in wastewater short-read metagenomics is shaped by background microbial abundance. **A) Background prevalence of representative pathogens.** Relative abundance distributions for *E. coli*, *C. difficile*, and *C. auris* across NSS and WWTP samples, with detection prevalence indicated for each taxon. **B) Spike-in experimental design.** Total nucleic acids from the three pathogens was spiked into extracted nucleic acids from NSS and WWTP samples (and pure water controls) at concentrations ranging from 10^−1^ to 10^5^ genome copies, followed by metagenomic library preparation and sequencing. **C–D) Detection responses to spike-ins.** Genome coverage (%) and relative abundance as a function of spike-in concentration for each pathogen in NSS (i) and WWTP (ii) backgrounds. **E) Conceptual model of detection sensitivity.** Illustration of how pathogen background abundance influences the ability of metagenomics based read-mapping and coverage approaches to enable detection from complex wastewater samples. Panels B and C are created in BioRender. Din, O. (2025) https://BioRender.com/.

Wastewater is a complex microbial matrix, and the ability of metagenomic sequencing to detect changes in specific pathogens depends in part on their background prevalence in the community. Accordingly, we investigated the baseline relative abundance and prevalence of clinically relevant human pathogenic species from reads mapping to *Escherichia coli*, *Clostridioides difficile*, and *Candida auris* using Woltka and NCBI’s RefSeq database (r 225). Short-read mapping against reference genomes revealed three distinct background burden profiles: *E. coli* was the most abundant (~1.0% of reads per sample, detected in all samples), *C. difficile* was detected at intermediate levels (0.0001–0.01% of reads, present in >99%), and *C. auris* was almost undetectable (0.00001–0.0001% of reads, present in only 2% of NSS samples) (Fig. 2A). These species thus served as representatives of high-, intermediate-, and low-background pathogens for subsequent detection testing.

To experimentally detect changes in pathogen species level abundance, we spiked nucleic acids from typical enteropathogenic *E. coli* (EPEC), *C. difficile*, and *C. auris* into extracted nucleic acid from both NSS and WWTP wastewater samples (Fig. 2B). By spiking in a range of genomic copies per organism, from 10^−1^ to 10^5^ copies – quantified via qPCR against standard curves using gBlock standards (Extended Data Fig. 2A-C), we sought to find the concentration thresholds at which these microbes could be detected for real-world application. Detection performance was evaluated using two readily achievable metrics from metagenomic data: percent genome coverage and relative read abundance. We found that *C. auris* possessed the least background interference when tracking spiked-in amounts using both percent genome covered and relative read abundance, although genomic coverage appeared more sensitive to changes in spike-in copies (Fig. 2C–D, i–ii). *C. difficile* spike-ins also produced discernable responses to increasing genome copies across both metrics with reduced resolution at lower spike-in levels; relative abundance was more sensitive to changes in spike-in copies. In contrast, spike-ins of typical EPEC did not produce discernable responses across either metric, even when mapping directly to a *de novo* assembled EPEC genome (Extended Data Fig. 2D-F) due to species-level abundance of *E. coli*. Results were consistent across NSS and WWTP sample types (Fig. 2C–D, i–ii). Thus, we identified a bifurcation for straightforward metagenomic detection against a complex background using relative abundance and simple genome coverage: the ability to continuously monitor pathogens by short-read shotgun metagenomics is greatly affected by the background of those species within the wastewater, where highly abundant genera present an ‘upper limit’ for the successful detection of short-term increases in burden, whereas low-background taxa can be tracked more sensitively (Fig 2E). These observations highlight the need for more advanced metrics for measurement of genomes from shotgun metagenomic data beyond these two approaches.

Combining these observations, we thus experimentally establish practical insights into the use of metagenomics as a method for pathogen monitoring. Furthermore, both genomic coverage and relative abundance can be successful in producing sensitive output, but the optimal choice may depend on the background prevalence and phylogenetic context of the target organism. Testing additional pathogens at different background levels and degree of phylogenetic dissimilarity from background relatives may therefore be an important direction for future study.

### Wastewater metatranscriptomics enables pathogen monitoring and elucidation of broad AMR diversity between NSS and WWTP samples

**Figure 3:**
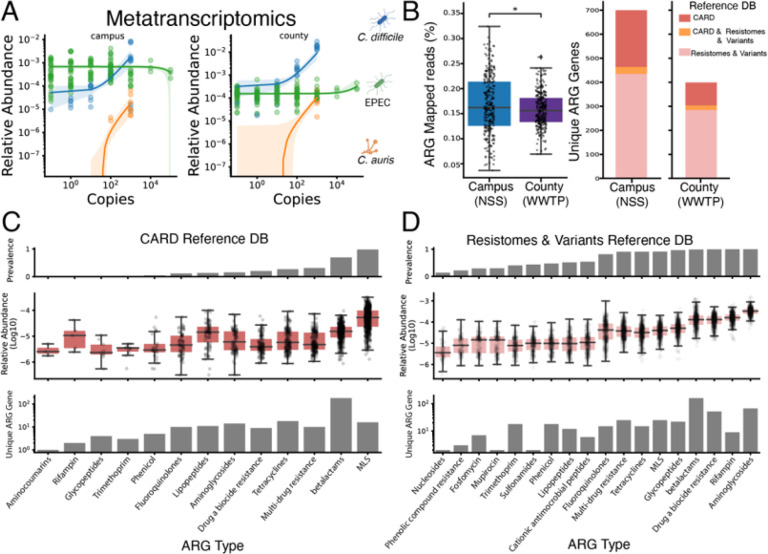
Metatranscriptomics enables detection of pathogens and AMR expression **A) Detection response of metatranscriptomic reads to pathogen spike-ins.** Relative abundance (log10) as a function of spike-in concentration for each pathogen in NSS and WWTP backgrounds. **B) Comparison of ARG detection across wastewater sample sources.** Boxplots (left) display distribution of percentages of ARG mapped reads across NSS and WWTP samples (P = 2.848 × 10^−2^, U = 3.683 × 10^4^). Countplots (right) of unique ARGs and their distribution among reference databases. Genes observed from metatranscriptomic reads aligning to distinct alleles, represented by either CARD or Resistomes & Variants reference DB, are displayed as a reference DB overlap (orange). **C-D) Description of ARG types in wastewater metatranscriptome samples, split by reference database.** Top barplots depict prevalence of ARG types among samples. Middle boxplots display log10 transformation of relative abundance of genes by ARG type. Bottom countplots (log10) tally number of unique genes of each ARG type.

Metatranscriptomic pathogen detection has potential advantages over DNA analyses as it enables detection of microbial function, including AMR gene expression. Therefore, we next sought to determine whether pathogen monitoring was feasible at the gene expression level. Using the same samples generated from the spike-in strategy outlined in Fig. 2B, we processed metatrascriptomics data to first remove ribosomal reads prior to read mapping analysis. We observed the same trends when analyzing transcripts for all three spike-in strains compared to the metagenomic data, with a higher limit of detection for *C. auris* (Fig. 3A). This pattern may be explained by differential effectiveness of particle capture of *C. auris* compared to the bacterial microbes. We again observed that the background RNA of *E. coli* was too high to detect EPEC spike-ins, with comparable results between both Campus and County samples (Fig. 3A). These data indicate that pathogen tracking is feasible with short-read metatranscriptomics, with similar limitations to short-read metagenomics. Furthermore, these results indicate that RNA datasets targeted at detecting RNA viruses may also be useful for analysis of bacterial and fungal pathogens in wastewater, despite the considerable potential for RNA degradation in that medium.

Because wastewater and downstream products of its processing are known to be hotbeds of AMR, we hypothesized that metatranscriptomics could further illuminate the differences between NSS and WWTP samples on AMR gene diversity. We compared the number of reads mapped to antimicrobial resistance genes (ARGs) using the Resistance Gene Identifier (RGI) and Bowtie2 alignments against the Comprehensive Antibiotic Resistance Database (CARD) of AMR genes including alignment against a Resistomes & Variants reference module of in silico predicted resistance variants^28^. Analysis of nonribosomal reads indicated that NSS Campus samples have a higher median ARG-mapped reads percentage than WWTP County samples (Mann-Whitney-Wilcoxon two-sided test, P = 2.848 × 10^−2^, U = 3.683 × 10^4^, Fig. 3B, left). More unique ARGs are identified from read mapping in the Campus samples than the County samples (Fig. 3B, right). These results indicate a loss of AMR gene expression from NSS to downstream WWTP samples, similar to the loss of microbial information between aggregates of the two sample types observed in Fig. 1.

To assess the impact of database choice on characterizing the AMR landscape in wastewater, we compared ARG type profiles generated using the canonical CARD database and the Resistomes & Variants database. The CARD database identified 13 ARG types, whereas the Resistomes & Variants database detected 16, with 11 types shared between the two (Fig. 3C–D, bottom). In the CARD analysis, macrolide–lincosamide–streptogramin (MLS) resistance was the most prevalent and abundant ARG type, consistent with prior reports of high macrolide resistance burden in urban wastewater metagenomes at a global scale^29^.

Relative abundances of shared ARG types were broadly similar across databases (Fig. 3C–D, center). However, in the Resistomes & Variants analysis, the four most abundant ARG types, shared by both databases, displayed higher relative abundances and were detected in a larger fraction of samples(Fig. 3C–D, top). These findings suggest that while both databases capture a similar AMR profile, incorporating AMR sequence variants, as in the Resistomes & Variants database, increases both the diversity and prevalence of ARG types recovered from wastewater metatranscriptomes. Further evaluation in diverse environmental contexts, beyond clinically derived datasets, will be essential to determine the robustness and interpretability of these expanded annotations.

### Long-read metagenomics of wastewater enables the identification of ARG-carrying microbial species

Although established short-read metagenomics and metatranscriptomics methods allow investigation of broad microbial taxa and AMR genes, particularly with MAG assemblies^15,23,30^, significant challenges exist in accurately capturing ARGs within the host context due to the dispersed nature of unique ARGs across many microbial species^13,31–33^. These data are thus not suitable for robust accurate species and strain level identification. To overcome these limitations, we applied long-read metagenomics to wastewater samples. Wastewater is a challenging sample type for obtaining large quantities of high-molecular weight (HMW) DNA. We found that gDNA produced by our experimental approach allows successful long-read metagenomic sequencing on the Pacific Biosciences (PacBio) Revio platform (see Methods)^6,34^, thus enabling one of the first long-read metagenomic investigations of wastewater.

**Figure 4:**
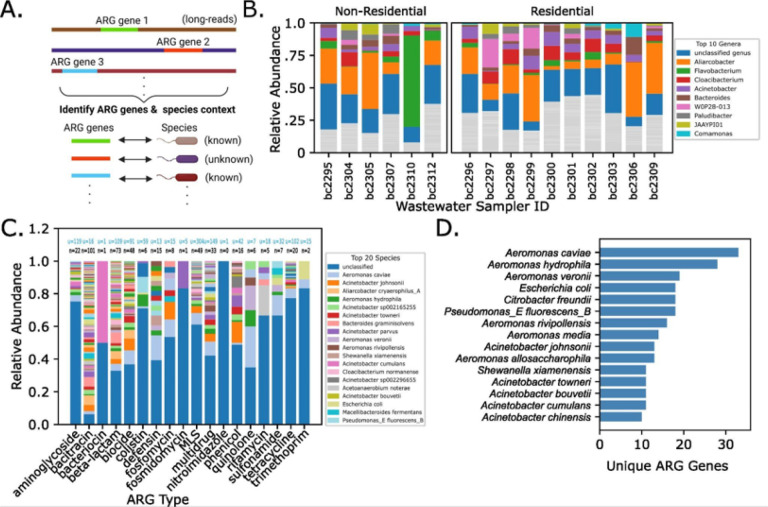
Long-read metagenomic reads enables species-level AMR gene context **A) Connecting ARG-genes to species-level context with long-reads.** Schematic of the analysis approach to delineate antibiotic resistance gene (ARG) information in species context using Argo. **B) Microbial genera across Campus samplers.** Taxa barplot showing the relative abundance at the genus level across near-source wastewater samplers. Colored taxa represent the top 10 genera across all samplers (see legend), while all other genera are in gray. **C) Distribution of microbial species across ARG types.** Taxa barplot showing the relative abundance of microbial species across identified ARG types. Species in the legend represent the top 20 species in terms of abundance across all ARG types. Above each bar, the number of classified species (black text) and unclassified species (blue text) are labeled. **D) Top ARGcontaining species.** Barplot showing the top 15 assigned species taxonomies in terms of number of unique ARG genes. Panel A is created in BioRender. Din, O. (2025) https://BioRender.com/.

While long-read metagenomics (LRG) has been demonstrated to produce complete genomes that are not achievable with short-read sequencing, we first test whether PacBio HiFi reads alone can illuminate the connection between AMR gene information and specific microbial species^35^. To accomplish this, we performed long-read sequencing on 16 samples from the NSS group, which had shown a higher number of unique ARG hits and microbial feature information in the short-read data (Fig. 4A). We developed a protocol that was capable of generating PacBio libraries with large fragment lengths resulting in a sequencing read length distribution with mean lengths of 8–10kb across all samples (Methods, Extended Data Fig. 3–4).

To identify ARGs within long-read metagenomic data and microbial species sequence context, we performed long-read AMR context analysis with the recently developed Argo tool^13,36^. We first analyzed the relative abundance of microbial taxa at the genus level, tracking their relative abundance across various wastewater samplers from both residential and non-residential buildings. Although the top genera observed were generally consistent across wastewater samplers and source type, we also found a high-abundance of unidentified microbial genera (Fig. 4B). To assess whether AMR genes classified by type were similarly affected, we examined species-level composition within ARG-associated microbes. Species representation varied considerably across ARG types, yet unidentified species were abundant in nearly every category (Fig. 4C). Interestingly, we observe that while there were similar numbers of unclassified ARGs known to be derived from genomic chromosomes, there were significantly more unclassified ARGs known to be derived from plasmids (Extended Data Fig. 5A). Thus, long-read metagenomic sequencing shows that unidentified ARG-carrying microbial species comprise a substantial portion of wastewater samples.

Multi-drug resistant microbes inherently possess many ARGs, therefore we next investigated which wastewater species possessed the highest number of unique ARGs. We analyzed the top 15 species in terms of unique ARG genes (Fig. 4D), and found that 11 of these species were also among the most abundant across ARG types, consistent with the idea that broad resistance is potentiated by many ARGs. Interestingly, of the top genera detected, four *Acinetobacter* spp., known to contain species with strong horizontal gene transfer potential and multidrug resistance, were identified. Also included were four *Aeromonas* spp., a genus which possesses many emerging human pathogen species^37^. Furthermore, we find an impressive 10–30 unique ARG genes in these species. *Aeromonas caviae* has over 30 unique ARGs, squarely within range of the average number of ARG genes found in the human pathogen *Acinetobacter baumannii*^38^. This is a significant observation given that *A. baumannii* is a priority target organism for the development of new antibiotics^39^, and that *Aeromonas* spp. are widely distributed in urban water and food sources (such as fish and poultry)^40–42^. These findings demonstrate that long-read metagenomics can discern species in wastewater that possess the highest AMR potential with known genomic context, especially in species that are of emerging clinical concern.

### Long-read metagenomics enables assembly of complete and novel culture-independent genomes from wastewater

**Figure 5:**
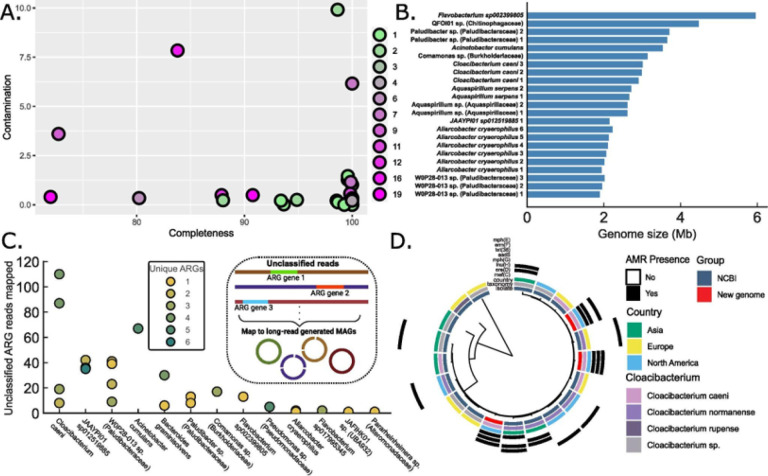
Long-read metagenomics produces full genomes of wastewater microbes **A) Complete and high-quality MAGs.** Plot showing the contamination and completeness of all the complete MAGs (MAGs, n=42) generated from long-read metagenomic assemblies. Each point represents one MAG, all less than 20 contigs. The color of each point indicates the number of contigs for each MAG (as shown in legend). Color gradient follows green to magenta for lower to higher numbers of contigs per MAG. **B) Single-contig, complete MAGs.** Barplot showing the length of the single-contig genomes (cMAGs) with identified taxa at the species, genus (shown as the genus name followed by sp.), or family level (shown in parentheses). Note that some taxa have multiple assemblies, such as *Cloacibacterium caeni*, denoted by a number for identification (1, 2, 3, etc..). **C) Long-read MAGs as sources of unclassified, ARG-containing reads.** Unclassified ARG reads identified in Fig. 4 mapped against the long-read *de novo* generated MAGs (inset). Plot shows the numbers of reads mapped to each MAG, colored circles, from its corresponding taxonomic classification represented on the x-axis. The color for each circle conveys the unique number of ARGs identified in the unclassified reads mapped to the MAG. **D) *C. caeni* comparison with closely related species.** Phylogenetic tree of marker genes rooted by midpoint of our newly assembled and publicly available genomes from *Cloacibacterium*. Tips are annotated by GTDB-Tk species identification and country of origin. Inset for panel C is created in BioRender. Din, O. (2025) https://BioRender.com/.

Having shown that unassembled long-reads can provide robust species context for AMR, we further investigated whether we could obtain genome-level assemblies to delineate strain information. We used the HiFi-MAG-Pipeline to first check contigs >0.5Mb for genome completeness, then subsequently assembled complete metagenome-assembled contigs (cMAGs)^43^ prior to binning, across 16 wastewater samples. We only selected MAGs with a minimal completeness of 70%, a maximum contamination of 10%, and a maximum of 20 contigs. Overall, we obtained 42 MAGs with 23 complete single-contig cMAGs, 7 high-quality MAGs (HQ-MAGs) at >90% completeness and <5% contamination, and 12 additional MAGs with >70% completeness and <10% contamination (Supplementary Table 3). We also observe that all MAGs possess less than 20 contigs, which is significant when compared to short-read MAGs which may possess hundreds of contigs (Fig. 5A)^43^. For the single-contig cMAGs, we observe broad similarity in genome sizes between different genomes assembled from the same species (Fig. 5B). Interestingly, we obtained 15 MAGs that classified to the genus (13 MAGs) or family (2 MAGs) level, with 9 of these being single-contig cMAGs. This observation highlights the capability for discovery of existing, but yet unknown, microbial species.

We then sought to determine if long-read assembled MAGs could be associated with any of the unclassified ARG-containing reads that we observed in Fig. 4. Across all samples, we were able to map ~18% of all unclassified ARG reads to 24 MAGs (13 of them single-contig cMAGs). The reads accounted for 1 – 6 unique ARG genes per genome, with numbers varying within classifications, indicating possible strain-level variation (Fig. 5C). Among the genomes with the most unclassified reads mapped, JAAYPI01 sp012519885 and W0P28–013 sp. possessed scant publicly available data. However, we observed the four genomes of *Cloacibacterium caeni* cumulatively possessing the highest number of unclassified ARG read hits. This species was first reported in 2017 and has been found to be prevalent in some wastewater sources, but little is known about its AMR potential^44,45^. To determine how AMR information in these genomes compared to publicly available data, we performed a pangenome analysis of the genus *Cloacibacterium*, where our genomes were phylogenetically clustered with strains previously isolated in Asia and Europe (Fig. 5D). Notably, our isolates carried a higher burden of ARGs in comparison to the public genomes, including mef(C), ere(D), lnu(H), and mph(G) — all of which had been previously observed only in related species from Asia (Fig. 5D, right). Overall, we identified 37 ARGs distributed across the 38 MAGs from our dataset by mapping all classified and unclassified ARG-containing reads (Extended Data Fig. 5b). These findings expand the known AMR gene repertoire in these groups and underscore their clinical relevance. We thus demonstrate that long-read assemblies can produce complete single contig genomes and HQ-MAGs, capable of capturing unique strain-level lineages and AMR properties, and expand our repertoire of microbial genomes for future comparative and evolutionary studies.

## DISCUSSION

Here, we present an approach to process wastewater samples for nucleic-acid based omics analyses, and carry out, to our knowledge, the first integrated study for untargeted microbial detection and microbiome analysis using a combination of shotgun metagenomics, metatranscriptomics, and long-read metagenomics on this sample type. Wastewater has gained prominence in recent years as an important medium amenable to the monitoring of microbial determinants of human disease. Prior wastewater microbiome studies have particularly focused on activated sludge, a downstream product of wastewater treatment^46^. In contrast, this study focused on near-source wastewater samples taken directly outside of buildings at UC San Diego and compared it to influent samples at San Diego County wastewater treatment plants., demonstrating that variability in microbial diversity is lost when comparing the campus samples. The two sampling strategies captured distinct microbial community structures: near-source sampling yielded greater heterogeneity, reflected by higher between-sample dispersion and greater within-sample diversity variance, whereas WWTP samples exhibited homogenized community profiles. This pattern is consistent with our hypothesis that wastewater collected upstream of sewer mixing retains variability arising from diverse human subpopulations and environmental inputs. Such heterogeneity may similarly characterize freshwater systems such as rivers or tributaries, where multiple sources converge downstream. Notably, non-residential NSS exhibited greater variability than residential NSS, likely reflecting transient and heterogeneous building populations compared with the more stable residential student communities. By contrast, WWTP influent displayed the lowest variability, likely due to extensive mixing and travel time across the sewer network before reaching treatment facilities. Future studies should expand these comparisons by systematically testing microbial enrichment strategies across wastewater types to evaluate their impact on the recovery and resolution of microbial diversity.

Pathogen detection in wastewater has primarily focused on targeted sensing of specific microbes in wastewater. We therefore addressed the amenability of both metagenomics and metatranscriptomics for non-targeted detection of specific pathogens at various levels of background within the wastewater. Using a variable pathogen spike-in approach, we determined that non-targeted detection of increasing levels of pathogen is possible with high accuracy. However, detection using typical mapping methods, as applied here, is limited by the background abundance of the species in the wastewater. Importantly, we demonstrate that untargeted metagenomics and metatranscriptomics can detect emerging pathogens which are not highly abundant in wastewater, such as *C. auris*^47^. Thus we established a preliminary bounds for the limits for the usability of metagenomics and metatranscriptomics for non-targeted detection of pathogens using readily achievable read-based mapping metrics. While more advanced computational approaches can help overcome these limitations, experimental approaches, such as biochemical depletion of abundant or non-pathogenic species, may serve as a complementary line of investigation to enable more robust pathogen detection.

Metatranscriptomics has been shown previously to be a useful tool to illuminate the diversity of AMR genes present within wastewater samples, which are known to be a hotbed of such genes^22^. Using this approach, we found that near-source campus samples possess more unique AMR genes than WWTP samples which aggregate signals from a whole county. Our data support the notion that near-source sampling is important to capture microbial wastewater compositions relevant to human health. Additionally, we determined that databases using ARG sequence variants analysis encompassed more information about ARG types, abundance, and prevalence. More studies are needed that utilize this technique as well as those that continue their development and refinement.

We also investigated the wastewater microbiome with long-read metagenomics^48^ using PacBio sequencing. We demonstrated that this approach can connect AMR genes to specific microbial species detected within wastewater. These results have profound implications in the potential ability to monitor multidrug resistant microbes which cannot be robustly detected with current short-read approaches or labor intensive culturomics. Additionally, these results can help identify species of interest for further strain-level investigation of horizontal gene transfer potential via study of extrachromosomal mobile genetic elements (eMGEs) using host–plasmid linkage (e.g., chromatin capture/proximity ligation based methods such as Hi-C or equivalent). Furthermore, we showed that many of the ARG-carrying species are unclassified, enabling this approach to robustly explore the ‘dark matter’ of ARG-carrying microbial species in wastewater. Importantly, we establish culture independent long-read metagenomics as capable of producing genome-level assemblies from a complex sample type. This approach can produce multiple genomes for the same species, yielding strain-level variation that can further fill our knowledge gap of within-species lineages. Furthermore, we can track novel AMR potential for these genomes by analyzing the presence of unique ARGs from existing genomes of related strains and also from taxonomically unclassified ARG-containing reads. Long-read metagenomics of wastewater opens up possibilities to dramatically expand our knowledge of specific microbial strains and their relevance to human health.

## METHODS

### Wastewater total nucleic acid extraction

Raw wastewater samples were stored at −80 °C following collection from campus NSS sites and WWTP influent pumping stations. After thawing, approximately 10 mL of each sample was transferred into 24-well plates containing 500 μl Nanotrap Microbiome A Particles (Ceres Nanosciences). A concentration step was performed on the KingFisher Flex Purification System (Thermo Fisher Scientific) to capture the microbial composition prior to extraction. The resulting eluate was transferred to a 96-well plate and total nucleic acids (NA) were extracted using the MagMax Microbiome Ultra Nucleic Acid Isolation Kit (Applied Biosystems). This approach of concentrating the samples prior to extraction allows for the genetic material for both of the viral and bacterial microbes from the sample to be extracted^49^.

### Contrived wastewater pathogen spike-in sample preparation

Stocks of *C. difficile* and *C. auris* were obtained from the American Type Culture Collection (ATCC). Typical EPEC strain E2348/69 O127:H6 was provided by the laboratory of Dr. Gail Hecht in collaboration with the AGA Fecal Microbiota Transplantation National Registry. These pathogens were transferred into 96-well racks of Matrix 1.0 mL ScrewTop tubes (Thermo Fisher Scientific) containing zirconia beads, and total NA was extracted using the MagMax Microbiome Ultra Nucleic Acid Isolation Kit (Applied Biosystems). This Matrix tube-based extraction method allows for high-throughput processing that is compatible with liquid handling automation and bead beating instruments^34^.

Because we chose to spike in extracted NA rather than titered live pathogens, a comparative qPCR analysis was performed to calculate the relative number of genomic copies present in each pathogen NA stock. TaqMan DNA primers and probes were selected or designed such that each qPCR assay targeted a unique genomic sequence specific to each organism. Additionally, corresponding gBlocks Gene Fragments (Integrated DNA Technologies) were designed to contain the DNA sequences being targeted in each assay. All qPCR assays were run in triplicate on the QuantStudio 7 Pro Real-Time PCR System (Thermo Fisher Scientific) using the Reliance One-Step Multiplex Supermix (Bio-Rad Laboratories). All primers, probes, and gBlocks were validated in both nuclease-free water (NFW) and wastewater backgrounds.

The gBlocks were diluted with NFW in a 1:10 serial dilution such that each qPCR reaction contained 10^6^ to 10^−1^ gene fragment copies, with 10^−1^ copies being equivalent to one copy in every 10 reactions. The resulting mean Cq values were plotted to generate a logarithmic standard curve for each target, to which the mean Cq values from reactions containing the extracted pathogen NA were fit to calculate the relative concentration of genomic copies in each NA stock. Additionally, this standard curve was used to determine the qPCR limit of detection for each DNA target, which was used to inform the spike-in concentrations. Pathogen NA stocks were serially diluted with NFW in a 1:10 dilution from 10^5^ to 10^−1^ copies per reaction; this range was selected to encompass one or more orders of magnitude above and below the qPCR limit of detection for each target. These dilutions were then spiked into the extracted NA from NSS and WWTP wastewater samples described above.

### Short-read library preparation and sequencing

Shotgun metagenomic and metatranscriptomic sequencing libraries were prepared from spiked-in wastewater total NA following a high throughput, miniaturized KAPA HyperPlus (Roche Sequencing Solutions) library preparation protocol, as previously described^50^. Briefly, double stranded DNA (or cDNA) from each sample was quantified using the Quant-iT PicoGreen dsDNA Assay Kit (Invitrogen), and then normalized to 5 ng in 3.5μL of molecular-grade water using an Echo550 acoustic droplet ejection liquid handler (Beckman Coulter Life Sciences). When sample DNA concentration was insufficient to reach the intended 5 ng input, a 3.5μL aliquot of sample was transferred. Enzymatic fragmentation (37 °C, 20 min), end repair/A-tailing, and adaptor ligation were performed at one-tenth reaction scale on a Mosquito HV (SPT Labtech). Adaptor ligation followed the iTru dual-indexing strategy^51^: universal “stub” adapters and ligase mix were added robotically, incubated at 20 °C for 30 min, and products purified with AMPure XP beads (Beckman Coulter Life Sciences) at 0.8× ratio using a BlueWasher (BlueCat Bio). Beads were resuspended in 6 μL water, and eluates combined with unique i5/i7 index primers in 10 μL PCR reactions containing 5 μL KAPA HiFi PCR MasterMix (Roche Sequencing Solutions) and 0.25 μL of each primer. Libraries were amplified for 15 cycles under manufacturer-recommended conditions and purified with paramagnetic beads as above. Purified libraries were eluted in 10 μL water, transferred to Echo-compatible plates, pooled in equal volumes, and assessed by TapeStation (Agilent Technologies). Pools were size-selected to 300–700 bp using Pippin Prep HT (Sage Sciences) and sequenced on an Illumina iSeq v2 (300 cycles) for initial quantification. A second, normalized sample pool was generated from sequence count normalization, as previously described^52^. Normalized pools were QC-checked, size-selected, and sequenced (2 × 151 bp) on a NovaSeq 6000 S4 flow cell v1.5 (Illumina).

Metatranscriptomic libraries were prepared by adding a cDNA synthesis workflow prior to the shotgun metagenomic protocol outlined above. Briefly, 30 μL of spiked-in wastewater total NA were treated with 1 μL TURBO DNase (Invitrogen) in 35 μL at 37 °C for 30 min in 96-well plates, followed by bead cleanup with RNAClean XP (Beckman Coulter Life Sciences, 2× ratio) and elution in 20 μL NFW using an epMotion 5075 (Eppendorf). Sixteen microliters of RNA were transferred via Bravo NGS liquid handler (Agilent) to a 96-well plate and four 96-well plates were compressed into a 384-well plate, vacuum dried (SpeedVac, Thermo Fisher Scientific), and resuspended in 2.5 μL NFW.

Bacterial rRNA was depleted at one-fifth reaction scale using QIAseq FastSelect 5S/16S/23S Kit (Qiagen). FastSelect master mix (500 nL) was dispensed via Mantis (Formulatrix) and incubated per manufacturer’s instructions. Ribo-depleted RNA was purified using QIAseq Beads (Qiagen, 1.3× ratio) with bead handling on a Mosquito HV and BlueWasher, and eluted in 6 μL NFW. Five microliters were transferred to a new 384-well plate, and samples were vacuum dried, followed by resuspension in 2 μL NFW.

First-strand synthesis using the SuperScript IV First-Strand Synthesis System (Invitrogen) was performed at one-fifth scale. RNA was primed with 200 nL dNTPs (10 mM each) and 200 nL random hexamers (50 ng μL^−1^) in a single 400 nL Echo 550 transfer. A 1.6 μL mastermix containing 200 nL DTT (100 mM), 200 nL RNase inhibitor (20 U μL^−1^), 200 nL SuperScript IV (200 U μL^−1^), 800 nL 5× first-strand buffer, and 200 nL actinomycin D (5 mg mL^−1^) was dispensed via Mosquito HV, followed by thermal cycling per manufacturer’s protocol.

Second-strand synthesis was performed by master mixing and adding 3 μL 5× Second-Strand Buffer (Invitrogen), 400 nL dNTPs (10 mM), 200 nL RNase H (2 U μL^−1^), 100 nL DNA ligase (10 U μL^−1^), and 100 nL DNA polymerase (Thermo Fisher Scientific) via Mosquito HV, backfilling to 15 μL with NFW, and incubating at 16 °C for 2 h. A final 1.6× bead cleanup (AMPure XP, Beckman Coulter Life Sciences) with a 80% ethanol wash was performed on the BlueWasher, and cDNA was eluted in 10 μL NFW (9 μL recovered) into an Echo-compatible plate for use as double-stranded cDNA input to the shotgun library preparation described above.

### Long read preparation and sequencing

Genomic DNA was quantified using the Qubit dsDNA BR Assay Kit (Invitrogen), and fragment length distribution was assessed using the Femto Pulse System (Agilent Technologies). Between 338 ng and 500 ng of gDNA was size-selected using the Short Read Eliminator (SRE) XS Kit (PacBio). Deviations from the manufacturer’s protocol included the use of a 96-well low-bind PCR plate, as well as the reduction of gDNA input volume and volume of SRE buffer used to 50 μL each, rather than the recommended 60 μL. This solution was subsequently centrifuged at 3,220 × g for 1 hour instead of the recommended 10,000 × g for 30 minutes. The resulting pellet was resuspended in 300 μL of Low TE buffer (PacBio). Size-selected DNA was reanalyzed on the Femto Pulse System before proceeding.

Shearing was performed using the SPEX SamplePrep 1600 MiniG homogenizer at 1,500 rpm for 1 minute and 30 seconds to generate ~10 kb fragments. Sheared DNA was processed using the HiFi prep kit 96 (PacBio) protocol, beginning at the post-shearing cleanup step. The final diluted AMPure PB cleanup and size-selection step was replaced with a 1× SMRTbell bead cleanup (PacBio). Final libraries were quantified using the Qubit dsDNA HS Assay Kit (Invitrogen) and analyzed on the Femto Pulse System. Two samples failed library construction and were excluded from sequencing. The remaining sixteen libraries had concentrations ranging from 0.2 to 2.06 ng/μL. A total of 170 pM of pooled libraries was loaded onto the PacBio Revio sequencing platform. The sequencing run yielded a total of 9.26 Gb of HiFi reads, with a mean length of 9.14 kb.

### Metagenomic and metatranscriptomic pre-processing and profiling

Following sequencing, metagenomic and metatranscriptomic reads were demultiplexed (bcl-convert[3.7.5], Illumina) and subject to stringent computational host filtration using a pipeline adapted from our previous work^49^ as implemented in Qiita^50^. First, quality filtering, adapter removal, and read trimming was performed using fastp^51^ (v. 0.20.1), then per-sample host reads were removed via consecutive alignment to human reference genomes GRCh38.p14^53^ and T2T-CHM13v2.0^54^ using minimap2^55^ (v. 2.28). For short-read analyses, minimap2 was run in short-read mode with parameter “-ax sr” and for long-read analyses, minimap2 was run in PacBio mode with parameter “-ax map-pb”. Finally, a pangenome index was created using Movi^56^ (v. 1.0) with the 94 currently available human reference genomes from the Human Pangenome Reference Consortium^57^ and reads with high-propensity pseudo-matching lengths to the human pangenome index were discarded. For short-read analyses, the default “custom” metric was used to account for both frequency and length of pseudo-matching lengths to the human pangenome reference. For long-read metagenomic analyses, the “max” metric was used with a threshold of 21. The resulting per-sample, host-filtered metagenomic reads were used for downstream analysis. Additionally, metatranscriptomic reads were fractionated into ribosomal and non-ribosomal reads using SortMeRNA^58^ (v2.1b). Downstream metatranscriptomic analyses were performed on the non-ribosomal reads fraction.

Per sample filtered short reads were processed in Qiita (Study ID: 15666, https://qiita.ucsd.edu/study/description/15666) using default metagenomic and metatranscriptomic processing workflows. For microbiome community analysis, metagenomic reads were aligned to the Web of Life database (release 2)^27^ using SHOGUN^59^ + Bowtie2^60^ (v. 2.4.2) in single-end mode(-q - --seed 42 --very-sensitive -k 16 --np 1 --mp “1,1” --rdg “0,1” --rfg “0,1” --score-min “L,0,−0.05” --no-head --no-unal), and translated to operational genomic unit (OGU) feature tables using Woltka^26^ v0.1.4. For pathogenic species detection, metagenomic and non-ribosomal metatranscriptomic reads were aligned to NCBI’s RefSeq database (release 225) using Shogun + Bowtie2 v2.4.2 in paired-end mode (--interleaved - --seed 42 --very-sensitive -k 16 --np 1 --mp “1,1” --rdg “0,1” --rfg “0,1” --score-min “L,0,−0.05” --no-head --no-unal --no-exact-upfront --no-1mm-upfront) and converted to feature tables with Woltka v0.1.7.

### Short-read microbial community diversity analysis

Multiple Qiita sample preparations (Study ID: 15666) were combined per data modality (metagenomic: 17371 + 17375, metatranscriptomic: 17372 + 17370) for the microbial community analysis. The feature space was filtered to include only OGU’s, reference genomes with alignment hits, that had genome coverage breadth of ≥ 1% across all samples. After filtering, the distribution of reads per sample of the full dataset, grouped by sample type, were evaluated to choose a rarefaction depth that maximized exclusion of BLANK library prep control samples and minimized wastewater sample dropouts. The metagenomics feature table was rarefied to 500 thousand reads and the non ribosomal metatranscriptomics feature table was rarefied to 30 thousand reads. Microbial community metrics of alpha and beta diversity were calculated using QIIME2(v2023.5). For alpha diversity, observed features and Faith’s Phylogenetic Diversity were calculated and statistical tests were performed with scipy (v1.8.1). Beta diversity was calculated using unweighted unifrac on and PERMANOVA statistical tests were performed with QIIME2(v2023.5).

To characterize differences in dispersion between college sites and between WWTP sites, leave-one-out convex hull volume assessment was performed as previously described^61^. The convex hull volumes were computed using q2-convexhull^62^ (v. 0.0.1) and represent the total volume enclosed by all the samples from each sampling site via PCoA of the unweighted UniFrac distance metric in the first three dimensions. To assess dispersion, the convex hull volume was computed per-site following removal of one sample and subsequent recomputation of the unweighted UniFrac distance using scikit-bio^63^ (v. 0.6.0), then this was repeated for every sample in each site to assess the stability of the convex hull volumes relative to sample-level variability. Differences in computed convex hull volumes were assessed using a Mann-Whitney U Test via scipy^64^ (v. 1.13.0). Scatterplots and boxplots were generated with matplotlib(v3.6.0), seaborn(v0.12.2), pandas(v1.5.3), and numpy (v1.23.5). Taxa set comparisons were visualized with UpSet(v0.9.0) and tree visualizations were created with EMPress(v1.2.0)^65^.

### Short-read pathogen detection

Metagenomic feature tables from alignment against RefSeq database (release 225) were collapsed to the species level and transformed to relative abundances in QIIME2 v2023.5^66^. Baseline relative abundances were evaluated for *Escherichia coli*, *Clostridioides difficile*, and *Candida auris* using histograms generated with Matplotlib, Seaborn, NumPy, and Pandas. Representative reference genomes in RefSeq varied by species, including one *C. auris* genome (GCF_003013715.1), five *C. difficile* genomes (GCF_030035795.1, GCF_000376285.1, GCF_000438845.1, GCF_020341515.1, GCF_018885085.1) and sixteen *E. coli* genomes (GCF_000350825.1, GCF_000019645.1, GCF_002853715.1, GCF_000008865.2, GCF_001281725.1, GCF_000263895.1, GCF_024519395.1, GCF_001865905.1, GCF_000690815.1, GCF_000734955.1, GCF_003697165.2, GCF_000010385.1, GCF_000210475.1, GCF_000005845.2, GCF_000459055.1, GCF_000013265.1). Prevalence was calculated as the proportion of samples with ≥1 read mapping to each species.

For spike-in detection, samples containing each pathogen at known concentrations were identified, and coverage percent and relative abundances were computed. Coverage percent, the fraction of the reference genome covered by at least one read, for *C. difficile*, *C. auris*, and EPEC was calculated using micov (v. 0.0.1). Coverage percent was then plotted against the spike-in concentration using the regplot function from seaborn (v. 0.13.2). The relative abundances for *C. difficile* and *C. auris* spike-ins were calculated at the species level, including all genomes listed above. Relative abundance, coverage percent, and mean coverage per bp for a *de novo* assembled EPEC genome was obtained by mapping reads via CoverM (v. 0.7.0). Paired regressions and scatterplots were generated using Matplotlib, Seaborn, NumPy, and Pandas.

### Long-read AMR analysis

AMR analysis of PacBio long-read data was performed using the recently developed tool, Argo (v. 0.2.1)^13^. Argo was made to use long-read data as input to perform ARG-profiling, then subsequently using these ARG-containing reads to perform taxonomic classification using Minimap2 and Diamond^55,67^. Reads which fail to be assigned to any taxon are labeled as ‘unclassified’. Additionally, Argo assigns the ‘unclassified’ label at a minimum genome-copy threshold. To mitigate unclassified labels based only on this threshold, we lowered this from the default of one genome copy (z=1) to z=0.25. Furthermore, for the genus-level taxonomy breakdown, we utilized the output of the initial profiling step, which uses Melon, a novel marker-based taxonomic profiler. For the species-level analyses, we utilize the output of the Argo pipeline, which only takes into account the ARG-containing reads.

Unclassified and classified long-reads were mapped to generated MAGs using minimap2 (v. 2.30) using the map-hifi flag. Hits are then parsed using primary alignments only with a minimum percent identity of 0.95 and minimum aligned fraction of 0.75.

### Long-read assembly and MAG analysis

Raw reads were converted from BAM to FASTQ and fasta format using bam2fastq (v. 3.1.1) and bam2fasta (v. 3.1.1), from the PacBio pbtk toolkit, respectively. The converted reads were assembled using Hifiasm-meta (v. 0.3) with default parameters, resulting in a high-quality metagenomic assembly for the wastewater samples^68^. The assembly was processed via the PacBio HiFi-MAG-Pipeline^43^ with to generate complete metagenome-assembled genomes (MAGs), which included binning via MetaBAT2 (v. 2.15) and SemiBin2 (v. 1.5), then comparison via DAS Tool (v. 1.1.6), and finally quality assessment of the genome via CheckM2 (v. 1.0.1)^69–71^. Quality filters included a minimum of 70% completeness, a maximum of 10% contamination, and a maximum of 20 contigs. Finally, taxonomic assignments were obtained with GTDB-Tk (v. 2.1.1, database version r207)^72^. Output summary files were concatenated into a single file for downstream analysis and used to generate relationships between contig numbers, completion, and contamination across all samples. Furthermore, MAGs generated by the pipeline were used for genome length, phylogenetic, and AMR analysis.

To construct a representative genomic dataset for analysis of assemblies, we combined all publicly available genomes from NCBI RefSeq (r229) corresponding to the taxonomic groups of interest with a set of newly sequenced isolates. Genome quality was assessed using CheckM2 (v1.0.1), and only genomes with ≥90% completeness and ≤5% contamination were retained for downstream analysis for both the publicly available and long read genomes. Gene prediction and functional annotation were performed with Bakta (v1.10.3) using default parameters^73^.

For pangenome construction of specific taxa of interest, we used Panaroo (v1.3.3) in strict mode to identify core and accessory genes across the genome set^74^. A multiple sequence alignment of the marker genes was generated within Panaroo and used to build a phylogenetic tree with FastTree2 (v2.1.11)^75^, RAxML (v8.2.12), and IQ-TREE 2 (v2.2.0.3). This tree was visualized and annotated with either the presence of antimicrobial resistance (AMR) genes, annotated with AMRFinder (v3.11.14), of interest or isolation metadata (e.g., country of origin) to highlight relevant epidemiological or functional patterns^76^.

## Supplementary Files

This is a list of supplementary files associated with this preprint. Click to download.

- DinetalSupplementalInformation.docx
- ExtendedDataFigures.docx

## Data Availability

*Raw sequencing data:* Shotgun metagenomic data and metatranscriptomics data are available on Qiita, Study ID#15666, and EBI, accessions PRJEB80377 and ERP164391. The spike-in typical EPEC genome is deposited on Zenodo (10.5281/zenodo.15061184).
